# Pregnancy Experiences of Female Sex Workers in Adama City, Ethiopia: Complexity of Partner Relationships and Pregnancy Intentions

**DOI:** 10.1111/sifp.12019

**Published:** 2017-03-06

**Authors:** Eileen A. Yam, Aklilu Kidanu, Brady Burnett‐Zieman, Nanlesta Pilgrim, Jerry Okal, Assefa Bekele, Daniel Gudeta, Georgina Caswell

**Affiliations:** ^1^ Associate, Brady Burnett‐Zieman is Staff Associate, and Nanlesta Pilgrim is Associate Population Council 4301 Connecticut Ave NW, Suite 280 Washington, DC 20008; ^2^ Director Miz‐Hasab Research Center Addis Ababa Ethiopia; ^3^ Associate Population Council Nairobi Kenya; ^4^ Adama Branch Manager and Daniel Gudeta is Project Coordinator Link Up, Organization for Social Services, Health and Development Addis Ababa Ethiopia; ^5^ Link Up, Organization for Social Services, Health and Development Addis Ababa Ethiopia; ^6^ Regional Advisor Link Up, International HIV/AIDS Alliance Cape Town South Africa

## Abstract

Research and programs for female sex workers (FSWs) tend to focus exclusively on HIV prevention, with little attention paid to how pregnancy affects their lives. We examine the circumstances surrounding pregnancy and childbirth among women selling sex in Ethiopia. In Adama City, researchers asked 30 FSWs aged 18 and older who had ever been pregnant to participate in in‐depth interviews. The women reported on pregnancies experienced both before and after they had begun selling sex. They identified some of the fathers as clients, former partners, and current partners, but they did not know the identities of the other fathers. Missed injections, skipped pills, and inconsistent condom use were causes of unintended pregnancy. Abortion was common, typically with a medication regimen at a facility. Comprehensive sexual and reproductive health services should be provided to women who sell sex, in recognition and support of their need for family planning and their desire to plan whether and when to have children.

Female sex workers are often referred to as a “key population” at elevated risk of HIV transmission because of their disadvantaged socioeconomic status, multiple sexual partnerships, and challenges with using condoms consistently. As a result, research and programs among this population typically focus exclusively on reducing HIV risk behaviors, with an emphasis on HIV testing and promotion of condom use. Comparatively little attention is paid to the reproductive health histories and fertility desires of women who sell sex, despite the fact that surveillance studies worldwide have found that most have children, and the vast majority have been pregnant (Scorgie et al. 2012). Ample evidence exists of unmet need for family planning and high abortion prevalence among women who sell sex, which has led to calls to integrate family planning into HIV programming for female sex workers (Petruney et al. 2012; Marlow, Shellenberg, and Yegon 2014; Schwartz 2015; Schwartz et al. 2015).

Not all FSWs aspire to space or limit pregnancy, however. Recent studies have found that a notable minority—about 10–15 percent—reported that they were trying to conceive with one of their partners, typically a nonpaying intimate partner (Beckham et al. 2015a; Schwartz 2015; Schwartz et al. 2015; Yam 2016). These findings underscore a fundamental reality for FSWs of reproductive age: not unlike their female peers who do not sell sex, sex workers’ decisions about whether and when to have children are dependent upon a range of individual, relational, and cultural factors. Programs for these women that solely emphasize increasing and measuring condom use run the risk of neglecting their basic family planning needs and fertility desires. This is particularly problematic for FSWs who are living with HIV, who often have limited access to prevention of mother to child transmission services or safer conception counseling (Schwartz et al. 2014; Schwartz 2015). This omission can ultimately lead to increased risk of vertical HIV transmission and unintended pregnancies among these vulnerable women (Beckham et al. 2015a; Beckham et al. 2015b; Schwartz 2015).

The complex relationship contexts in which FSWs experience pregnancy create substantial challenges to researchers who seek to examine their fertility desires and family planning behaviors across multiple partners. The traditional reliance on quantitative behavioral surveillance studies among FSWs is not always conducive to understanding the heavily context‐dependent nature of pregnancy among women whose relationship status, fertility desires, and disease‐prevention behaviors can vary dramatically across sexual encounters and partners. Unlike respondents to traditional demographic surveys—which typically assess contraceptive method use in the context of women's relationships with a single man—women who sell sex often report different contraceptive use and fertility desires across multiple men.

In recent years, a small number of studies have described how FSWs navigate parenthood while selling sex. A recurring theme in these studies is that FSWs’ parenting roles play a critical role in their decisions to initiate and continue selling sex. Their need to provide for their children is a major motivation for engaging in sex work, and many struggle to balance child care with their work (Zalwango et al. 2010; Basu and Dutta 2011; Beckham et al. 2015b; Duff et al. 2015). However, far less is known about the specific contexts in which these women experience pregnancy. One qualitative study from Tanzania sought to understand FSWs’ experiences with intended pregnancy, concluding that many saw childbearing as a way to earn respectability as a mother or to solidify intimate relationships, even if conceiving meant engaging in risky sexual practices. Expanding on this previous research on FSWs’ intended pregnancies, our article describes their experiences with pregnancy more generally—including intended as well as unwanted or unplanned pregnancies—among gravid FSWs in Adama City, Ethiopia. Specifically, we aim to explore intendedness of the pregnancies, use of contraception at the time of pregnancy, and pregnancy outcomes (e.g., miscarriage, stillbirth, abortion).

## CONTEXT

Our study was conducted as part of the Link Up project, a global sexual and reproductive health and rights consortium led by the International HIV/AIDS Alliance focused on young key populations. Ethiopia was one of five countries in which Link Up activities were implemented. In 11 cities, local and international nongovernmental organizations implemented peer outreach programs to provide HIV and sexual and reproductive health and rights education and services to young people (aged 10–24) who were living with HIV, young people selling sex, and other vulnerable youth. The Link Up project also trained health care providers at participating clinics to provide comprehensive, nonjudgmental HIV and sexual and reproductive health services to vulnerable young people.

Adult HIV prevalence in Ethiopia is 25 percent higher among women compared with men (1.9 percent versus 1.5 percent), and according to a 2011 study approximately 23.8 percent of Ethiopian FSWs are living with HIV (Federal Democratic Republic of Ethiopia 2014). Since Ethiopian FSWs were among the beneficiaries of Link Up service delivery and advocacy activities, this study was conceptualized to explicitly explore and describe the circumstances surrounding their pregnancies. The site for the current study was Adama City, Ethiopia where research partner Miz‐Hasab Research Center had previously conducted a behavioral survey of venue‐based FSWs (i.e., those who sell sex in brothels, hotels, or other establishments). Based on findings from that earlier study, approximately one‐third of FSWs in Adama City had children, among whom 14 percent had two or more (Mooney et al. 2013). This article contributes a more nuanced understanding of the reproductive histories of Adama City FSWs, beyond a simple quantification of their parity.

Sex work is not explicitly prohibited under Ethiopian law, and women sell sex relatively openly and freely in cities and towns (Overs 2013). In a country with one of the highest HIV prevalence rates in the world, FSWs are regarded as a high‐risk population in Ethiopia, with an estimated 24 percent living with HIV (UNAIDS 2014). Unintended pregnancy is common among Ethiopian FSWs, many of whom have children. One recent study that examined family planning needs among FSWs in Ethiopia took place in Mekelle city and found that 29 percent of the women had experienced an unintended pregnancy in the past two years, among whom 60 percent had had an abortion (Weldegebreal et al. 2015). In a five‐city study of Ethiopian FSWs, 38 percent of participants had children (Girma and Erulkar 2009).

## METHODS

### Study Design and Data Collection Procedures

Between August and October of 2014, trained research assistants conducted qualitative in‐depth interviews of 30 FSWs who were participating in peer outreach activities through local nongovernmental organization and Link Up service delivery partner, Organisation for Social Services, Health and Development (OSSHD),^1^ in Adama City. We determined that a sample size of 30 would be sufficient to achieve saturation in themes and to achieve study aims, based on general guidelines recommended by social science and public health researchers. Research assistants approached women attending OSSHD programs at the OSSHD facility and invited them to participate in a study, asking them brief screening questions to determine eligibility. We purposively sampled women who participated in OSSHD programs, were age 18 and older, had ever experienced pregnancy, and reported sex work as their main source of income.

Before their interviews, all participants were given a choice of providing oral or written consent to take part in the study, depending on their literacy and comfort levels signing their names. No identifying information was collected from participants. Trained research staff conducted the one‐on‐one interviews in a private space on‐site at the OSSHD office. Open‐ended interview questions solicited information about participant demographic characteristics, reproductive health and HIV prevention behaviors, contraceptive use, and experiences as a mother engaged in sex work. Interviewers used a semi‐structured interview guide that included a series of questions designed to describe the pregnancy history for each woman. These questions probed each pregnancy that participants reported experiencing, including those that did not result in a live birth. For example, the pregnancy history included open‐ended questions about who the fathers were for each pregnancy, whether the pregnancy took place before or after initiating sex work, whether the woman was using contraception at the time, and whether the pregnancy was intended. The interviews were conducted in Amharic, on‐site at the OSSHD office, audio‐recorded with the women's permission, and subsequently transcribed and translated into English for analysis.

The analysis involved three stages in order to develop the codebook and identify key themes. During the first stage, the lead author prepared an initial list of codes and definitions as well as preliminary themes, informed by the existing literature on pregnancy and motherhood among FSWs. In the second stage, using this preliminary codebook, three analysts coded the same eight transcripts with the qualitative data analysis software Atlas.ti. These eight transcripts were selected to include at least one transcript from each interviewer, as well as at least two participants who reported living with HIV. Upon comparing the three analysts’ coded transcripts, the lead author calculated the interrater reliability coefficient for the three sets of codes, which suggested substantial agreement across the three coders (kappa = 0.6). In consultation with the coders, the lead author refined the codebook content and definitions to address and reconcile any discrepancies across the three coders, as well as add any new themes that arose in the eight transcripts. In the third analysis stage, each of the remaining 22 transcripts were divided between the three coders and thematically analyzed and coded using the finalized codebook (Boyatzis 1998). In addition, the lead author reviewed every transcript and tabulated the general context and outcomes of all the pregnancies that the participants reported on. For this article, the research team focused on key themes that pertain to participants’ experiences with pregnancy, family planning, and abortion. Among women who voluntarily self‐reported that they were living with HIV, we also examined the timing of diagnosis relative to pregnancies, as well as access to prevention of mother‐to‐child transmission services. To examine differences between pregnancies experienced before women entered sex work compared with pregnancies after sex work initiation, the lead author employed a constant comparative method (Corbin and Strauss 2008) that assessed whether the same concepts were identified for pregnancies experienced before and after sex work initiation.

This study was reviewed and approved by two ethical review committees: the Institutional Review Board of the Population Council in the United States, and the Oromiya Regional Health Bureau's Health Research Ethical Review Committee in Ethiopia.

## RESULTS

The 30 participants ranged in age from 18 to 35, with a median of eight years of education. Thirteen (43 percent) had an intimate partner, and one of these women was married. Eleven women (37 percent) had been pregnant once, 19 (63 percent) two or more times, and three (10 percent) were currently pregnant. Six (20 percent) reported having no children. Twenty‐one women (70 percent) were using a nonbarrier female‐controlled modern contraceptive method, defined as the pill, injectable, or implant. Nearly half had terminated a pregnancy. Four (13 percent) reported that they were living with HIV (Table 1). The cumulative pregnancy histories of the 30 participants encompassed 66 total pregnancies.

**Table 1:** Participant characteristics (N = 30)

	N (%)
Age—median (range)	23 (18–35)
Years of education—median (range)	8 (4–10)
Has intimate partner^a^	13 (43)
Number of pregnancies	
1	11 (37)
2	10 (33)
3+	9 (30)
Currently pregnant	3 (10)
Number of children	
0	6 (20)
1	14 (47)
2+	9 (30)
Uses nonbarrier modern contraception^b^	21 (70)
Ever had an abortion	14 (47)
Self‐reported living with HIV	4 (13)

a One of these 13 women was married.

b Pill, implant, or injectable.

### Pregnancy Intentions: Influenced by Past Pregnancy Outcomes and Partner Desires

Nearly half of participants described at least one of their past pregnancies as intended. Their reasons for wishing to get pregnant at the time were to provide older children with a sibling, to provide their partners with a child, and to make up for the loss of a previous pregnancy that was not carried to term. One 23‐year‐old participant—whose first pregnancy had ended in stillbirth—described her second pregnancy as intended, remarking, “I had to forget my first pregnancy's memory.” Similarly, another 23‐year‐old who had previously had an abortion described her third pregnancy as intended, “since I got rid of the last one and [my partner] wanted a child.” As another example of how previous pregnancy outcomes influenced women's subsequent desire for children, one 22‐year‐old participant stated, “I had two daughters and I wanted a boy so I had one.”

Several women framed their decision to get pregnant in terms of their partners’ desire for children. Likewise, among women who had husbands or partners at the time of pregnancy, some mentioned that their partners wanted them to get pregnant, and others described childbearing as their fundamental duty as married women.

If it's a marriage, having children is a responsibility. [FSW, age 20]

I had a plan to make [my lover] my own by giving him a child. [FSW, age 24]

### Circumstances Surrounding Unintended Pregnancies

Five women said that they first got pregnant when they had sex for the first time, before they began to engage in sex work. In all cases, the fathers were husbands or partners, and they did not use any protection. Furthermore, several commented that they had little to no knowledge of contraception or pregnancy at the time. A 19‐year‐old who got pregnant the first time she had sex (at age “15 or 16”) said that the father was her “school boyfriend” at the time, and that he never found out that she has his child.

In one case, a woman said she got pregnant when she had unprotected sex with her first client at age 18. However, among women who experienced an unintended pregnancy after they had started selling sex, in most cases they said that the father was a partner or boyfriend at the time.

Some women also suggested that sexual violence played a role in their unintended pregnancies. Two women volunteered that they had gotten pregnant as a result of rape. One woman was raped by the son of her boss when she was 18 years old, and the other said that a client “used force” to have sex with her, leaving her pregnant.

### Contraception at the Time of Pregnancy: Inconsistency, Misinformation, and Method Failure

At the time of most of the reported pregnancies, women were not using any contraception, despite the fact that nearly all participants said they knew where they could obtain family planning methods. One 20‐year‐old woman who was experiencing a first‐time pregnancy stated at the time of the interview: “I didn't use birth control methods for the last two years… and I thought I would not get pregnant since nothing [had ever] happened to me in this regard.”

Several women said that the month they got pregnant they had forgotten to take their pill or injectable and were, therefore, not protected from pregnancy. Others held misconceptions about how to use their contraceptive methods effectively, or reported that they got pregnant when the condom broke during sex.

I forgot to get injected. Five days had already been passed from the day that I had to get injected. Then I started to get nauseated. And I went for pregnancy checkup and I was told that I was pregnant. [FSW, age 19]

I have heard that if you use injections for a long time, you can interrupt taking the injection and not become pregnant for about one year. That was not true in my case. I became pregnant in less than two months. [FSW, age 26]

The condom broke and that time I didn't use any type of contraceptives. For that, I got pregnant. [FSW, age 24]

### Identities of Fathers: Diversity of Relationships

A majority of the women could identify the men with whom they got pregnant. In most cases, the fathers were partners or husbands at the time of pregnancy. Several participants reported that they got pregnant with clients, and in most cases they knew the identities of the fathers. Some of these men supported the children once born, providing some financial support.

However, women did not always know the identities of the fathers. Two women volunteered that they had gotten pregnant by unknown fathers, and others simply described the father as “a client,” without elaborating on whether they knew their identity. One of the women, age 22, who did know the father's identity recalls that the condom broke one time during the month that she got pregnant, so she assumed his paternity. The other woman, age 28, also did not know the father's identity nor was she certain whether she was using a condom at the time: “I didn't remember [if I used a condom]. The condom might have broken.”

### Selling Sex while Pregnant

Among women who became pregnant after their entry into sex work, several reported continuing to sell sex late into pregnancy, sometimes into the third trimester. In all cases, they said that they continued to work during pregnancy out of financial necessity. Explained one 28‐year‐old who worked three months into her pregnancy: “I had to make money for survival.”

Several women spoke about how, despite being visibly pregnant, there were still clients who would pay them for sex. These women described the varied reactions from their clients, ranging from sympathy to insensitivity.

I was very anxious. I did not want them to press me hard. Some clients understand my feelings, but some others don't. [FSW, age 20]

Some of them did not care. Some would feel sorry for you, give you some money and walk away. Some ask you why you are doing this while pregnant. So, you just have to deal with different situations. [FSW, age 26]

### Abortion Experience: Very Common, Sometimes Unsafe

In 2004, the Ethiopian penal code was reformed to loosen restrictions on abortion. The law now allows abortion under a wide range of circumstances, including: the health or life of mother or fetus is in danger, cases of rape or incest, fetal abnormalities, or when the woman is a minor unprepared to raise a child (Criminal Code of the Federal Democratic Republic of Ethiopia 2005; Ipas 2010). In theory, the Ethiopian abortion law is relatively permissive—permitting minors to obtain abortions, for example—but in practice, access to abortion remains inadequate, and 6 out of 10 abortions are unsafe (Singh et al. 2010; Center for Reproductive Rights 2015). Fourteen of the 30 women interviewed had ever terminated a pregnancy, including two who had had three or more abortions each. In most cases, they underwent a surgical or medication abortion procedure at a facility, most frequently mentioning commercial or nongovernmental organization clinics. These women reported paying fees ranging from 100–1,000 birr (US$5–$50).^2^

Despite the relatively permissive legal climate for abortion, four women described their experiences attempting to terminate pregnancies with traditional providers and methods—sometimes aiming to avoid the fee that a clinic would charge for abortion services. Due to either unsuccessful abortions or post‐abortion complications, three of those women ended up subsequently receiving post‐abortion care at an NGO clinic or hospital.

The procedure was really painful and it took days after days…. I was about to die and friends contributed money and send me to [a nongovernmental organization clinic] and they thought I was going to die, but I survived. [FSW, age 22]

### Pregnancy among Female Sex Workers Living with HIV

Of the four women who disclosed that they were living with HIV, two became pregnant after they were diagnosed. One of these women's children acquired HIV through mother‐to‐child transmission. The pregnancy was unintended, resulting from unprotected sex with the man who had fathered one of her other children. She had not yet initiated sex work at the time of this pregnancy. Newly aware of her HIV status, she had her newborn tested and the child, too, was HIV‐positive.

One of my children is now nine years old and she lives with the virus. This is because I gave birth at home and breastfed her without knowing anything. [FSW, age 35]

One woman who was living with HIV reported that she was diagnosed before her first and only pregnancy, predating her initiation into sex work. She initiated antiretroviral treatment during pregnancy, and her child received a negative HIV test upon birth.

[During an antenatal care visit], I gave blood and then the result was HIV‐positive. I told the doctor that I knew my result before and I came to receive the treatment to prevent mother‐to‐child HIV transmission. She gave me the medicine and I went home. [FSW, age 19]

### Comparisons between Pregnancies before and after Initiating Sex Work: Commonalities and Differences

Of the 30 participants, 13 reported that all the pregnancies they had experienced took place before they first started selling sex. An additional 7 women experienced pregnancy both before they first engaged in sex work, and after. To explore whether the previously described themes and findings differed between pregnancies experienced before and after first engaging in sex work, we conducted a separate thematic analysis that examined the 32 pregnancies that took place prior to initiating sex work, compared to the 34 pregnancies experienced after the women started selling sex. It is worth noting that, among pregnancies that took place after women first started selling sex, it was not always possible to ascertain whether women were earning income as a sex worker at the time of each pregnancy; there may have been some fluidity with which women entered and exited the sex trade several times throughout their lives.

With the exception of findings regarding selling sex during pregnancy—which is only relevant to those who were working in the sex trade at the time of pregnancy—the findings regarding contraceptive use (or lack thereof), pregnancy intendedness, abortion experience, and circumstances surrounding pregnancy were similar, regardless of whether pregnancies took place after starting sex work or not. Furthermore, there were pregnancies in both categories that resulted from rape, unprotected first sex, and fathered by husbands or partners. One noteworthy contrast was that, among pregnancies that occurred before starting sex work, all of the women knew the identities of the fathers; this was not universally the case among pregnancies experienced after initiating sex work, as described previously. Nevertheless, pregnancies with unidentified fathers were comparatively rare, and the most commonly mentioned paternal identities were husbands or partners, even among women who had entered sex work. In addition, condom breakage was only cited as a factor among pregnancies that took place after sex work initiation. Table 2 lists the frequency with which selected themes arose in women's reports of pregnancies before and after sex work initiation.

**Table 2:** Context and outcomes of pregnancies experienced before and after sex work initiation (N=66)^a^

	Pregnancies before sex work initiation (n = 32)	Pregnancies after sex work initiation (n = 34)
No contraception used when she got pregnant	14	10
Method misuse (e.g., missed pill or injection)	4	8
Condom breakage	0	4
Intended pregnancy	8	7
Induced abortion	4	11
Paternal identity unknown	0	2

a Women did not always report on each of these circumstances for every pregnancy, and at times the information was equivocal. For example, it was not always clear if a father described simply as a “client” was a known individual to the woman, so we err on the side of regarding these as cases of a known paternal identity. This table only includes those instances when a woman explicitly stated that the circumstance applied to her pregnancy. Therefore, the frequencies reported should not be interpreted as proportions, but rather a potential illustration of the salience of each of these themes among those pregnancies that women opted to describe in depth.

## DISCUSSION

This study complements the existing quantitative research regarding FSWs’ family planning needs and behaviors. Unlike survey data—which often are restricted to fairly limited closed‐ended questions regarding parity or contraceptive use, for instance—we attempt to elucidate the specific context surrounding each of the pregnancies that participants experienced. By encouraging participants to sequentially recount the circumstances surrounding pregnancy within the broader context of their lives—including a reflection on the timing of those pregnancies relative to their entrance into sex work—this analysis attempts to address knowledge gaps that have not been addressed by many previous cross‐sectional studies on this topic (Sloss and Harper 2004; Reed et al. 2013; Beckham et al. 2015a; Beckham et al. 2015b; Papworth et al. 2015). Based on the reproductive histories of the female sex workers in Adama City, it is evident that one cannot ascribe to these women a static, singular identity as a sex worker throughout their reproductive lives. In contrast, in our analysis we found that there were many women who only experienced pregnancy with husbands or partners in the years *preceding* their entry into sex work. Many began selling sex years after first becoming mothers. Nevertheless, it was also quite common for women to describe pregnancies that occurred *after* they had begun selling sex—intended and unintended, with clients and with intimate partners.

In terms of intendedness, contraceptive use, or pregnancy outcomes, the contexts of pregnancies prior to entrance into sex work were not notably different from those that women experienced after beginning to sell sex. These commonalities support the contention of scholars and advocates who call for a more holistic, rights‐based approach to FSWs’ reproductive needs (CHANGE 2016); the mere fact that women sell sex does not mean that their desire for children or wantedness of pregnancies should necessarily be different than those of women who do not sell sex. However, whereas women could report the identities of the fathers of all the pregnancies that took place before sex work initiation, for a small number of pregnancies that occurred after sex work debut, the identities of the fathers was unknown. After initiating sex work, it is to be expected that women have a broader range of sexual partnerships, encompassing clients with whom they had less intimate—and sometimes anonymous—relationships. As articulated cogently by Beckham and colleagues (2015a), “While female sex workers and other women of reproductive age share many pregnancy experiences, unique circumstances also exist among female sex workers, given the nature of their work” (p. 56). The diversity in the fathers among women selling sex is the critical difference between pregnancies that took place before and after initiating sex work. By inquiring explicitly about the timing of pregnancies relative to women's initiation into sex work, we were able to examine the wide diversity of circumstances under which they got pregnant throughout their life spans.

These findings support previous studies that have demonstrated that women who sell sex do not receive sufficient family planning information or services (Scorgie et al. 2012). Despite recent calls to integrate HIV and family planning services in programs for female sex workers (Schwartz et al. 2015)—often by broadening service offerings to include provision of family planning counseling and nonbarrier modern contraception—we are aware of no such integrated efforts that have been evaluated or that prioritize measuring impact on family planning outcomes (such as unintended pregnancy incidence or contraceptive discontinuation). Unfortunately, given the primacy of HIV funding and HIV‐related outcome indicators in sex worker programming, add‐on family planning services may be given short shrift in individual counseling interactions. As a first step, to ensure that women's broader family planning needs are addressed, future integrated programming should explore the feasibility and impact of introducing routine, provider‐initiated family planning needs assessments, rather than relying on clients to proactively inquire about family planning. Such universal screening will need to explore women's potentially divergent childbearing intentions across the full spectrum of sexual partners, taking care to acknowledge and respect their right to decide whether and when to have children.

Consistent with previous studies, participants’ accounts of their pregnancy histories revealed the substantial diversity in women's relationships with the fathers. Likewise, the extent to which women planned to get pregnant varied dramatically by partner as well as by pregnancy. Women spoke about how their desire for children was influenced by the outcomes of previous pregnancies, particularly in cases where earlier pregnancies ended in induced abortion. Their sense of obligation to have children also was manifested in their statements about how they got pregnant expressly because their partners wanted them to. For several women, having children was a woman's essential duty in a marriage. This centrality of motherhood to FSWs’ identities as parents and as wives has been documented in previous studies (Basu and Dutta 2011; Beckham et al. 2015b).

Consistent with previous studies among FSWs, abortion was very common among participants (Weldegebreal et al. 2015; Willis, Welch, and Onda 2016). Compared with FSWs who have received abortions in more legally restrictive contexts (Marlow, Shellenberg, and Yegon 2014; Madeiro and Diniz 2015), women in our study mostly terminated pregnancies with a skilled provider, typically using a medical abortion regimen. Based on their accounts, most participants who wished to have abortions were able to access safe abortion care. Although there was a notable minority who attempted to terminate pregnancies using traditional remedies or providers, most of them ultimately accessed post‐abortion care in facility settings.

A significant limitation of this study is that our participants were limited to FSWs who were accessing OSSHD services and outreach. Arguably, these women have better access to information and services compared with their peers who are not receiving such services. It is likely that the broader FSW community in Adama City has more pronounced reproductive health needs. A second limitation is the variability in the extent to which participants provided detailed contextual information about each of their pregnancies, such as the timing of pregnancies relative to initiation of sex work, the nature of the relationship with the fathers, or the intendedness of the pregnancies. Some women divulged greater details than others, which may be a result of variation in interviewer styles as well as differences in participants’ desires to share their deeply personal stories. Furthermore, given time and resource constraints, we decided not to purposively sample a larger number of women who reported living with HIV. As a result, there were only four participants who could speak to their experiences as HIV‐positive sex workers, which is too small a sample size to ensure that the thematic analyses achieved saturation for this subgroup.

Program implementers face substantial barriers to meeting FSWs’ reproductive health needs: an entrenched societal aversion to regarding FSWs as pregnant women or mothers, the “siloed” nature of HIV and reproductive health programming and financing, and the challenges of balancing FSWs’ disease prevention needs with their childbearing desires (Schwartz et al. 2014; Beckham et al. 2015a; Schwartz 2015). Ideally, FSW programs’ traditional emphasis on HIV testing and condom promotion could be broadened to also include provision of—or referral to—family planning, safe abortion care (where permissible), and quality maternal health care services. Despite recent calls for greater integration of HIV and sexual and reproductive health services (Schwartz et al. 2015), programs for FSWs often fall short of this goal. Health care providers and programs that serve FSWs should be sensitized to the fact that women who sell sex experience pregnancy under diverse circumstances, including unprotected first sex, misuse of contraception, sexual violence, as well as both intended and unintended pregnancies. Furthermore, programs must be responsive to how many sex workers have very limited access to health care services, and they confront deeply entrenched stigma and discrimination, which can further constrain access to care. Traditional family planning providers need to be sensitized to the highly complex, often fraught dynamics between sex workers and their sex partners, with whom consistent condom use may not be a viable option. Though they represent a small proportion of the population, the holistic sexual and reproductive health needs of FSWs should be met in a coordinated, integrated fashion, with an emphasis on upholding their fundamental right to plan whether and when to have children.

## Footnotes

1 At the time of the fieldwork, OSSHD was the Organisation for Social Services for AIDS (OSSA). The organization changed its name in early 2016.

2 US$1 = 20.28 ETB as of February 2015.
